# Lipomatous apocrine adenoma with syringocystadenoma papilliferum arising from the external auditory canal

**DOI:** 10.1186/1758-3284-3-36

**Published:** 2011-08-22

**Authors:** Tzu-Cheng Su, Ko-Hung Shen, Hsin-kai Wang, Pei-Yi Chu, Mei-Ling Chen

**Affiliations:** 1 Department of Surgical Pathology, Changhua Christian Hospital, Changhua, Taiwan; 2 Jen-Teh Junior College of Medicine, Nursing and Management, Department of Medical Technology, Miaoli, Taiwan

**Keywords:** tubular adenoma, syringocystadenoma papilliferum, external auditory canal

## Abstract

A case of lipomatous tubular adenoma (LTA) with syringocystadenom papilliferum (SCAP) arising from the external auditory canal in a 25-year-old man is described and to the best of our knowledge through literature review, this kind of morphologic entity has not been reported before. Herein we reported the first case in the English literature in the world.

## Introduction

Tumors arising from the external ear canal are extremely rare. Exostosis and squamous cell carcinomas are the most commonly-encountered benign and malignant tumors, respectively. Tubular adenoma (TA), also named as tubular apocrine adenoma, tubular syringoadenoma, or tubulopapillary hidradenoma [1,2], was first reported as a new entity by Landry and Winkelmann in 1972 [3]. Since then, less than one hundred cases of tubular adenoma were reported in the English literature [4]. This type of rare tumors was reported at the scalp, extremity, and eyelid [5]. Tubular adenoma originated in the external auditory canal was first reported by Lee and his colleague in 2005 [6]. In Lee's report, he described a tubular apocrine adenoma with syringocystadenoma papilliferum. Here we described the first case of a lipomatous tubular adenoma with syringocystadenoma papilliferum arising from the external auditory canal.

## Case report

A 25-year-old male was referred for hearing impairment and discharge on and off from left ear for a period of time. Family history is unremarkable and laboratory data is with normal limit. Pure tone audiometry evaluation was arranged and conductive hearing loss of left ear was identified. A mass noted in the left external auditory canal. High resolution computed tomography showed an approximately 0.8 × 0.6 cm soft tissue mass occupying within the left external ear canal near the opnening, without eroding canal wall. Tumor excision was performed and the resected tumor was submitted for pathological examination. The patient recovered uneventfully without evidence of recurrence nine months after operation.

## Pathological findings

**Figure 1:**
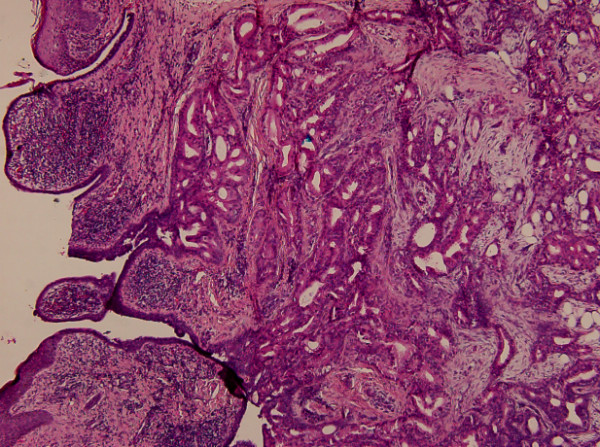
**Histologic examination shows two different portions of tumor**. The left lesion of the tumor shows syringocystadenoma papilliferum with papillomatous projection into the invaginations of the surface epithelium. The right lesion of the tumor reveals lipomatous tubular adenoma composed of double-layered tubular structure. (H&E, original magnification × 40).

**Figure 2:**
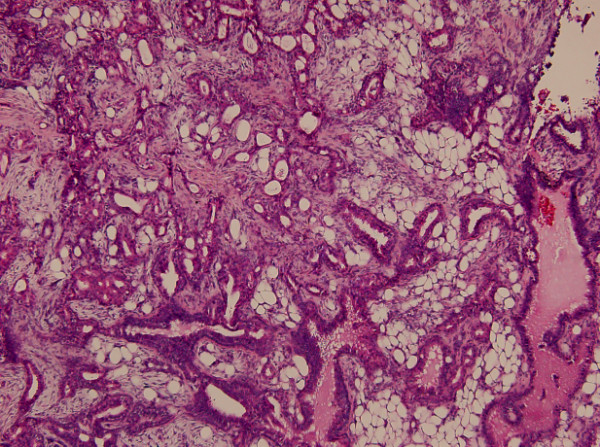
**The stroma of lipomatous tubular adenoma is composed of hyalinized fibrous tissue with intervening abundant adipocytes**. "Decapitation" secretion is seen. (H&E, original magnification × 100).

**Figure 3:**
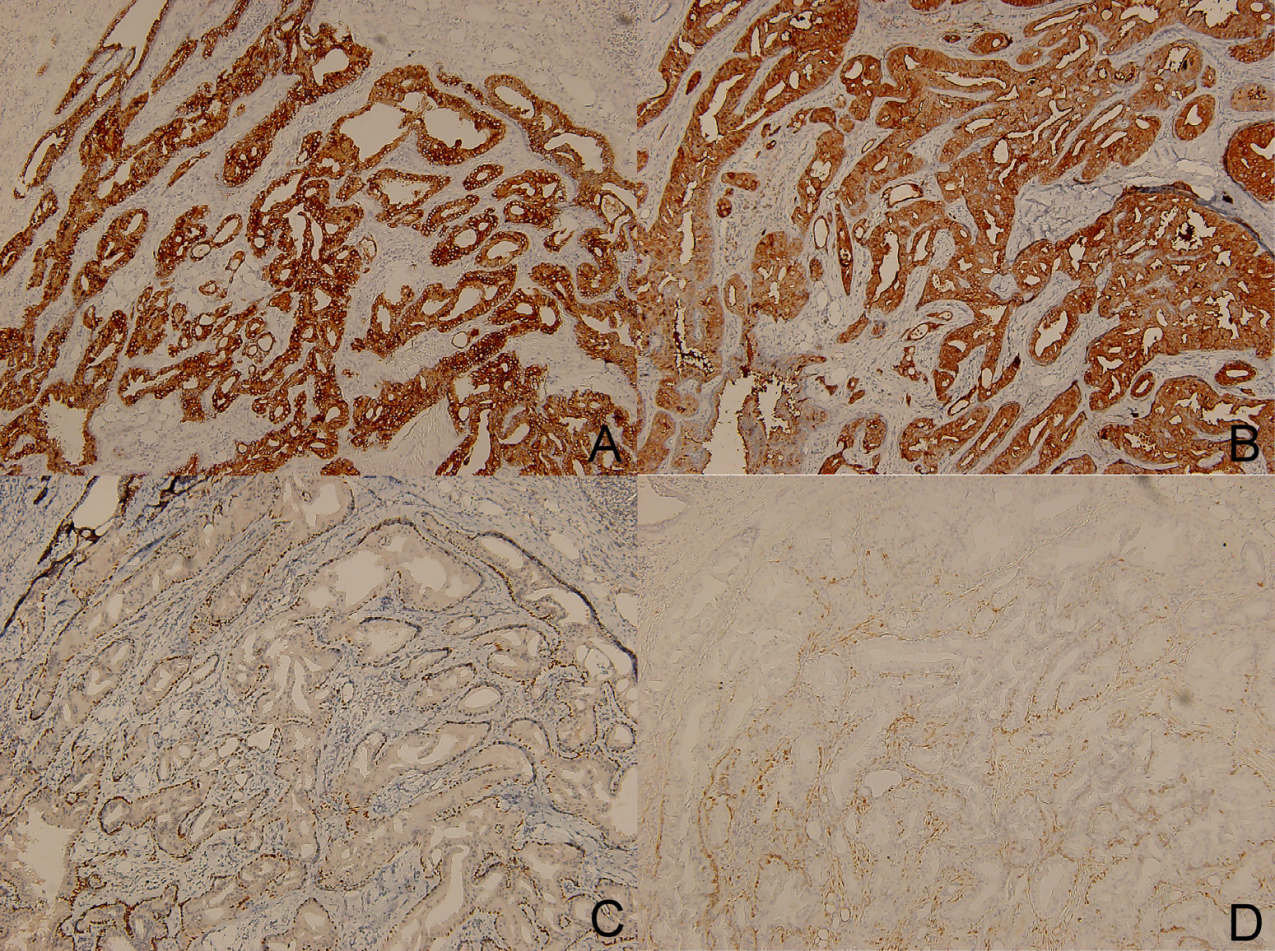
**Immunohistochemical studies revealed bilayer different lining cells**. Positivity for cytokeratin-7 (Figure 3A) and GCDFP-15 (Figure 3B) in the inner layers of cylindrical cells of the tubules were shown. Positivity for p63 (Figure 3C) and S-100 protein (Figure 3D) in the outer layers of cuboidal or flattened cells with myoepithelial differentiation were shown. (Immunohistochemical stain, original magnification × 100).

Section of the tumor shows circumscribed lobules of well-differentiated double-layered tubular structures located in the deep dermis (Figure 1). The stroma is composed of fibrous tissue with intervening abundant adipocytes (Figure 2). The inner layers of cylindrical cells of the tubules have apocrine features showing "decapitation" secretion (positive for cytokeratin-7 and GCDFP-15) (Figure 3A and 3B). The tubules are surrounded by the outer layers of cuboidal or flattened cells with myoepithelial differentiation (positive for P63 and S-100) (Figure 3C and 3D). Abnomal mitotic figure, invasive border, and cellular atypia are not noted. No inflammatory cells are noted in the tumor. Lipomatous tubular adenoma is diagnosed. Section of the upper part of the tumor shows features of syringocystadenoma papilliferum with irregular papillary projections protruding into the invaginations of the surface epithelium. Transition from squamous epithelium to double-layered cuboidal and columnar epithelium is seen. Plasma cells admixed with lymphocytes is noted in the stroma. No nevus sebaceous is seen in the background of the tumor.

## Discussion

TA is a rare entity and sometimes occurs with SCAP. SCAP was previously reported to be often associated with pre-existing nevus sebaceous [7], and morphologic features of nevus sebaceous seldom existed in SCAP with TA [1]. No nevus sebaceous is seen in our case. Controversy about the relationship of TA and SCAP still exists. TA was considered as a minor variant of SCAP by some scholars [8], but others have suggested that TA is a distinct clinical entity [7]. In Kazakov's recently published study, 67 cases of TA, SCAP, and their lookalikes were assessed by four dermatopathologists. Only 29 cases got concurrent agreement and interobserver variation existed in other 38 cases.

TA or SCAP was reported to have certain relationship with human papillomavirus infection [9] and a human papillomavirus induced non-neoplastic process leading to TA or SCAP was also speculated by some authors [1]. Warty surface or koilocytotic feature is not seen in our case.

Immunohistochemical study may be useful in puzzling cases. S-100 protein was reported to be detected in the peripheral myoepithelial cells in the TA and failed to be present in the SCAP [7,10], which was consistent with the immunohistochemical results of our presented case.

Treatment of LTA with SCAP includes careful preoperative evaluation and surgical resection with safe margin. Computed tomography is useful in evaluating the tumor and surrounding tissue.

## Conclusion

Our case shows the characteristic SCAP and TA with abundant adipocytes in the upper and the deeper portion, respectively. In a thorough research for PUBMED, this is the first reported case with LTA and SCAP occurring in the external auditory canal. Although Lee at al [6] published the first case of TA occurring in the external auditory canal, but abundant adipocytes are mixed with fibrous tissue in the stroma of TA in our case, and this kind of morphologic presentation has not yet reported in the external auditory canal.

## Consent statement

Written informed consent was obtained from the patient for publication of this Case report and any accompanying images. A copy of the written consent is available for review by the Editor-in-Chief of this journal

## List of abbreviations

LTA: Lipomatous tubular adenoma; SCAP: syringocystadenom papilliferum; TA: tubular adenoma.

## Competing interests

The authors declare that they have no competing interests.

## Authors' contributions

TS: wrote, aligned and drafted the manuscript. KS participated in the collection of the data, literature review. HW: collected clinical and pathological records. PC: took the pictures for this manuscript. MC: modified this manuscript and make and final approval.

All authors read and approved the final manuscript.
